# Profiles of attitudes toward inclusive education among Chinese in-service teachers: their relationships with demographic factors and organizational commitment

**DOI:** 10.3389/fpsyg.2024.1391862

**Published:** 2024-05-15

**Authors:** Yuhao Deng, Wei Yuan, Li-fang Zhang, Zhengli Xie

**Affiliations:** ^1^Institute of Education, University College London, London, United Kingdom; ^2^School of Education, Central China Normal University, Wuhan, China; ^3^Faculty of Education, The University of Hong Kong, Hong Kong, China; ^4^Faculty of Humanities, The Hong Kong Polytechnic University, Hong Kong, China

**Keywords:** attitudes toward inclusive education, organizational commitment, Chinese in-service teachers, latent profile analysis, special educational needs

## Abstract

**Introduction:**

The increased diversity of students (e.g., students with special educational needs) has presented teachers with unprecedented challenges. Teachers’ attitudes toward inclusive education play a crucial role in teachers’ organizational well-being. However, existing studies mostly explored attitudes toward inclusive education based on a variable-centered approach. This study used a person-centered approach to identify teachers’ attitude profile membership and explored the relationships of attitude profiles with demographic factors (i.e., gender, years of teaching experience, subject taught, and in-service training) and organizational commitment.

**Methods:**

Nine hundred and seventy-two in-service teachers from forty-nine inclusive education schools in Beijing responded to the Revised Multidimensional Attitudes toward Inclusive Education Scale and the Organizational Commitment Inventory. Latent profile analyses, multinomial logistic regression, and univariate analysis of variance were used to analyze data.

**Results and discussion:**

The results revealed four attitude profiles: *involuntary participation*, *behavior avoidance*, *neutral*, and *proactive involvement*. Years of teaching experience and in-service training were significant predictors of teachers’ latent profile membership. Teachers belonging to the *involuntary participation* profile showed the highest levels of maladaptive commitments to inclusive education schools. Teachers belonging to the *proactive involvement* and the behavior avoidance profiles showed higher levels of adaptive commitments. However, teachers belonging to the *neutral* profile had the lowest levels of adaptive commitments. The theoretical contributions, practical implications, and limitations are discussed.

## 1 Introduction

Inclusive education became a worldwide trend in education reform after more than ninety countries signed the Salamanca Statement and Framework for Action in 1994 [United Nations Educational, Scientific and Cultural Organization (UNESCO), 1994]. Regular education schools have been undergoing a comprehensive and radical restructuring into the so-called inclusive education schools (Loreman et al., 2014). Among a variety of school factors, scholars have consistently argued that teachers’ attitudes toward inclusive education (ATIE) are the key determinants of effective inclusive education (Ewing et al., 2018; Van Steen and Wilson, 2020). Teachers in favor of inclusive education provide individualized support for students with special education needs (SEN), help typically developing students to accept and interact with their SEN peers, and collaborate with special educators (Jordan et al., 1997; Yang, 2005; Yeo et al., 2014); and thus, their students with SEN in inclusive education schools can achieve desirable academic progress and social development. However, teachers with negative ATIE rarely interact with their students with SEN, rarely apply teaching methods known to be successful in inclusive environments, and are reluctant to collaborate with other special education professionals (Bender et al., 1995; Tiwari et al., 2015). Students with SEN thus feel isolated and some even move to special education schools (Fu and Xiao, 2016).

The importance of teachers’ ATIE has made it an intensely researched topic in the past decades. A wealth of research has been conducted in various countries using a quantitative variable-centered approach. This approach assumes that all individuals within a sample are a homogenous group (Ferguson et al., 2020) in determining teachers’ ATIE types (i.e., positive, negative, and neutral). For example, Slovenian teachers held positive ATIE (Štemberger and Kiswarday, 2018), while Finnish teachers displayed negative ATIE (Saloviita, 2020). Neutral ATIE were found among Serbian teachers (Galović et al., 2014). Compared with findings from quantitative studies, qualitative studies have identified more complicated attitudes among teachers of students with SEN. For instance, through focus group interviews among primary school teachers in Singapore, Yeo et al. identified two ATIE groups: a positive group (e.g., satisfaction and happiness) and a negative group (e.g., frustration, fear, and anxiety) (Yeo et al., 2014). Adopting the hermeneutic phenomenology approach, Tiwari et al. found Indian teachers showing three types of ambivalent attitudes: (a) high willingness to teach students with SEN but without actual actions, (b) adequate understanding of inclusive education principles but denying the meaning in actual practice, and (c) investing much effort but disagreeing to accept students with SEN in regular classes (Tiwari et al., 2015). These complicated results indicate that a single classification (e.g., positive or negative) identified using quantitative variable-centered approaches is inadequate for explaining teachers’ ATIE. Although qualitative research results are enlightening, they have often been criticized for being unrepresentative because of their small sample sizes.

This study adopted latent profile analysis (LPA) to explore teachers’ ATIE to overcome the limitations using quantitative variable-centered approaches and qualitative approaches. LPA is one of the quantitative person-centered approaches that aim to identify hidden groups from data by examining the probability of individuals belonging to different groups (Ferguson et al., 2020). Compared with variable-centered approaches, LPA can categorize teachers into diverse groups, within which, members have similar ATIE characteristics. According to the review of previous findings (Yeo et al., 2014; Tiwari et al., 2015), it is believed that teachers’ ATIE can be divided into heterogeneous groups. Compared with qualitative methods, the results of LPA are generated from a relatively big sample size based on scientific indicators (for further details, see Section 2.3). Furthermore, LPA enables researchers to determine how the identified profiles predict or are predicted by other factors (Ferguson et al., 2020). In studies using a variable-centered approach, teachers’ demographic factors were found to influence their ATIE (see Section 1.2 for more details). It is unknown whether or not ATIE profiles would be influenced by demographic factors. Therefore, one of the aims of this study was to examine the influence of teachers’ demographic factors on their profile membership.

Organizational commitment refers to individuals’ psychological contract with their work organization (Meyer and Allen, 1991). Against the backdrop of school-wide inclusive education reforms in which the ways (e.g., teaching methods) and the contents (e.g., developing an individualized education plan for SEN students) of teachers’ work have been changed, teachers with an adaptive organizational commitment to their schools are vital for the success of inclusive education reform (Xie, 2022). Adopting a variable-centered approach, Xie and Zhang found that teachers’ ATIE statistically contributed to organizational commitment (Xie and Zhang, 2022). However, little is known about the relationships between different teachers’ ATIE profiles and organizational commitment. Understanding these relationships could help change teachers’ ATIE profiles to become aligned with their schools’ mission to develop inclusive education, which also enhances their psychological commitment to the schools. Therefore, this study also examined the differences in organizational commitment among teachers with different ATIE profiles.

### 1.1 Theoretical framework for teachers’ attitudes toward inclusive education

The three-component (i.e., cognitive, affective, and behavioral) theory of attitudes (Eagly and Chaiken, 1993) is a widely known theoretical framework for understanding people’s opinions about an object (e.g., a thing, person, or idea) (de Boer et al., 2012). Based on the three-component model (Eagly and Chaiken, 1993), Mahat developed the Multidimensional Attitudes Toward Inclusive Education Scale (MATIES) to explore Australian teachers’ ATIE (Mahat, 2008). The MATIES’s cognitive component concerns teachers’ views and beliefs related to inclusive education; its affective component involves teachers’ emotions and feelings about inclusive education; and its behavioral component reflects teachers’ intentions to act in a particular manner toward inclusive education 20. The MATIES was subsequently used to examine teachers’ ATIE in other cultural contexts, including Scotland (MacFarlane and Woolfson, 2013), India (Srivastava et al., 2015), Slovenia (Štemberger and Kiswarday, 2018), and Ghana (Butakor et al., 2020). The three components (i.e., cognitive, affective, and behavioral) of the MATIES were adopted in this study as a theoretical framework for exploring teachers’ latent ATIE profiles. However, the three-component model for teachers’ ATIE did not fit the data well in several studies (Butakor et al., 2020) and the MATIES has not been used previously in mainland China. Therefore, the MATIES was revised and validated in this study before LPA was performed.

### 1.2 Demographic factors influencing teachers’ attitudes toward inclusive education

Studies using a variable-centered approach highlighted the importance of teachers’ demographic factors (e.g., gender, in-service training, and years of teaching experience) influencing their ATIE. For example, in-service training focused on inclusive education has been recognized widely as a determinant of teachers’ ATIE. More positive attitudes were often found among teachers participating in such training (de Boer et al., 2011; Srivastava et al., 2015). In terms of years of teaching experience (abbreviated as ‘teaching experience’ hereafter), studies have shown a negative relationship between teachers’ teaching experience and their ATIE. For instance, Butakor et al. found that teachers with <3 years of teaching experience showed more positive attitudes than teachers with 3–10 years of teaching experience (Butakor et al., 2020). Teachers with 3–10 years of teaching experience held more positive attitudes than those with >20 years of teaching experience (Butakor et al., 2020). Similarly, Kuyini et al. found that the ATIE of teachers who had been teaching for 1–5 years was more positive than the ATIE of teachers who had been teaching for 6–10 years (Kuyini et al., 2020). Gender differences in teachers’ ATIE have also been found in the literature. For example, Romi and Leyser discovered that female teachers’ ATIE were more positive than that of male teachers (Romi and Leyser, 2006). In the Chinese context, subject taught is a vital demographic factor in the psychological attributes of teachers working in inclusive education settings. For example, core subject (i.e., Chinese language, mathematics, and English) teachers had lower levels of self-efficacy in teaching students with SEN than general subject (e.g., music, art, and physical education) teachers (Xie et al., 2022). Whether or not there are subject taught differences in teachers’ ATIE remains unknown. Thus, the subject taught was examined in this study. Overall, four demographic factors, that is, gender, teaching experience, in-service training, and subject taught, were selected in this study to examine whether they statistically influence teachers’ ATIE profile membership.

### 1.3 Theoretical framework for organizational commitment

Meyer and Allen’s three-component (i.e., affective, normative, and continuance commitment) model is the best known theoretical framework for organizational commitment (Meyer and Allen, 1991). Based on this three-component model, Ling et al. constructed a five-component model of organizational commitment for the Chinese context (Ling et al., 2002). The five components are affective commitment (emotional attachment to the organization), normative commitment (obligation to the organization), choice commitment (difficulties in finding other jobs), economic commitment (fear of economic loss because of leaving the organization), and ideal commitment (occupational aspirations). Studies have suggested that affective, normative, and ideal commitment have adaptive value because they are positively related to adaptive personality traits (e.g., openness), creativity-generating teaching styles, and positive emotions in teaching, while economic and choice commitment have maladaptive value because they are positively related to maladaptive personality traits (e.g., neuroticism), norm-favoring teaching styles, and negative emotions in teaching (Zhang, 2015; Zhang and Jing, 2016; Zhang, 2019). Therefore, this study examined the differences in adaptive (i.e., affective, normative, and ideal) and maladaptive (i.e., economic and choice) commitments among teachers with different ATIE profiles.

Overall, this study had four objectives: (1) to validate the Revised-MATIES in Chinese inclusive education contexts; (2) to identify latent profiles in teachers’ ATIE based on their Revised-MATIES score; (3) to investigate the influence of demographic factors (i.e., gender, teaching experience, subject taught, and in-service training) that may determine teachers’ latent profile membership; and (4) to examine the differences in organizational commitment among teachers with different ATIE profiles.

## 2 Methods

### 2.1 Participants and procedure

Before data collection, ethics approval was obtained from the Human Research Ethics Committee of the University of Hong Kong. Then, the online consent form, demographic information sheet, and the two inventories measuring ATIE and organizational commitment were designed using the Wenjuanxing platform^1^ to collect data. In the consent form, participants were informed of the purpose and significance of this study. To protect their privacy, they were also informed that no personal data would be identified or disclosed and their participation was entirely voluntary. They could terminate this survey at any time without any negative consequences. If they were willing to participate in this survey, they could select the buttons “I agree to participate in this study” and “Next page” to start the formal survey. All collected data were stored in a password-protected hard drive and locked in a cabinet in the corresponding author’s office.

The online survey was sent to forty-nine Beijing primary schools that implemented inclusive education. A total of 998 teachers of students with SEN in regular primary schools participated in this study over a 1-month period. Nine hundred and seventy-two questionnaires were validated. Among the 972 teachers, 163 were men and 809 were women. Six hundred and fifteen had attended in-service training on inclusive education, while 357 had not. Five hundred and sixty-five were core subject teachers and 407 were general subject teachers. Four hundred and eighty-seven teachers had teaching experience between 1 to 15 years and 485 had >16 years of teaching experience. Their ages ranged from 23 to 59 years (*M* = 38.75, SD = 8.58).

### 2.2 Measures

#### 2.2.1 Revised multidimensional attitudes toward inclusive education scale (MATIES)

The MATIES is an 18-item inventory consisting of three components: cognitive (6 items), affective (6 items), and behavioral (6 items) (Mahat, 2008). The MATIES has not been used previously in mainland China; therefore, it was translated and adapted before beginning this study. First, one author who is a native Chinese speaker and fluent in English translated the MATIES into Chinese. Second, a doctoral candidate who is a native English speaker and fluent in Chinese performed back-translation. Third, all authors discussed the wording of each item together. Considering that several items in the cognitive component of the MATIES did not fit the data well in earlier studies (Butakor et al., 2020); therefore, the authors modified three items from the cognitive component. Specifically, Item 1 ‘I believe that an inclusive school is one that permits academic progression of all students regardless of their ability’ was changed to ‘I believe that it is difficult for students with a disability to achieve academic progress in inclusive schools.’ Item 3 ‘I believe that inclusion facilitates socially appropriate behavior among all students’ was changed to ‘I believe that it is difficult for inclusive schools to facilitate socially appropriate behavior for students with a disability.’ Item 4 ‘I believe that any student can learn in the regular curriculum of the school if the curriculum is adapted to meet their individual needs’ was changed to ‘I believe that it is difficult to adapt the regular curriculum of the schools according to the needs of students with a disability.’ Fourth, two professors in the field of inclusive education were invited to review all items and provide feedback. After discussion and modification, the Revised-MATIES was finalized. The participants were requested to rate themselves on a 6-point Likert scale (1 = strongly disagree and 6 = strongly agree). The negatively stated items were reversely coded. The higher the teachers’ scores, the more positive the attitudes they held toward inclusive education.

#### 2.2.2 Organizational commitment inventory (OCI)

The 17-item Organizational Commitment Inventory was adopted to measure the teachers’ organizational commitment (Zhang, 2015). It measures five organizational commitment components: affective (3 items), normative (3 items), ideal (5 items), choice (3 items), and economic commitment (3 items). The participants were asked to rate each item on a 5-point Likert scale (1 = not applicable at all and 5 = very applicable).

### 2.3 Data analysis

The data were analyzed with SPSS 26.0 and M*plus* 7.4 (Muthén and Muthén, 1998–2012). First, exploratory factor analysis, confirmatory factor analysis, and Cronbach’s α values were performed to test the psychometric properties of the Revised-MATIES and the OCI. Second, latent profile analyses were performed with one to five profile solutions. Following Howard et al., Akaike information criterion (AIC), Bayesian information criterion (BIC), sample-size adjusted BIC (ABIC), Lo–Mendell–Rubin likelihood ratio test (LMR), bootstrap likelihood ratio test (BLRT), and entropy were used to determine the best solution (Howard et al., 2016). Lower AIC, BIC, and ABIC values suggest a better model fit (Ferguson et al., 2020). The LMR and BLRT values were used to compare the improvement in fit between the k-profile and the k − 1-profile. A significant *p*-value indicates that the k-profile model improves the fit over the k − 1-profile model (Ferguson et al., 2020). Entropy values of >0.80 indicate good classification accuracy (Tein et al., 2013). Third, multinomial logistic regression was used to examine the influence of demographic factors (i.e., gender, teaching experience, subject taught, and in-service training) on the likelihood of attitude profile membership. The odds ratio (OR) was used to indicate the effect size. Finally, univariate analysis of variance (ANOVA) and *post hoc* follow-up tests were performed to examine how the four attitude profiles differed in terms of the five organizational commitment components.

## 3 Results

### 3.1 Psychometric properties of the instruments

The Kaiser–Meyer–Olkin (KMO) measure of sampling adequacy and Bartlett’s test of sphericity were conducted on the Revised-MATIES before running the exploratory factor analysis. The results suggested that the KMO value was 0.93 (*p* < 0.001, approximate *χ*^2^[153] = 10,978.57), which indicated that factor analysis was suitable for the data (Kaiser, 1974). Exploratory factor analysis using principal axis factoring with promax rotation was performed to test the factor structure of the Revised-MATIES. The results yielded three factors with eigenvalues >1 (see Table 1). All 18 items loaded on the theoretically expected factors (i.e., the cognitive, affective, behavioral components) and the three factors accounted for 67.43% of the variance in the data. The confirmatory factor analysis results supported the three-factor model of the Revised-MATIES (Hu and Bentler, 1999): *χ^2^* = 880.66, *p* < 0.001, *df* = 132, Tucker–Lewis index (TLI) = 0.92, comparative fit index (CFI) = 0.93, root mean square error of approximation (RMSEA) = 0.08, and standardized root mean square residual (SRMR) = 0.05.

**Table 1:** Exploratory factor analysis for the Revised-MATIES.

Item	Factor 1	Factor 2	Factor 3
1	0.87		
2	0.86		
3	0.83		
4	0.79		
5	0.79		
6	0.73		
7		0.82	
8		0.80	
9		0.78	
10		0.74	
11		0.71	
12		0.70	
13			0.82
14			0.81
15			0.80
16			0.77
17			0.75
18			0.61
Eigenvalue	7.45	2.94	1.75
Cumulative variance	41.39%	57.73%	67.43%

Factor 1 = behavioral component; Factor 2 = affective component; Factor 3 = cognitive component.

The confirmatory factor analysis results also supported the five-component model of organizational commitment. The model fit indices for the OCI were *χ*^2^ = 442.31, *p* < 0.001, *df* = 109, TLI = 0.97, CFI = 0.98, RMSEA = 0.06, and SRMR = 0.04. The model fit indices were satisfactory (Hu and Bentler, 1999).

The reliability of the Revised-MATIES was 0.90 for cognitive component, 0.89 for affective component, and 0.91 for behavioral component. The reliability of the OCI was 0.93 for affective commitment, 0.86 for normative commitment, 0.94 for ideal commitment, 0.89 for choice commitment, and 0.80 for economic commitment. Thus, the reliability of both the Revised-MATIES and OCI was good (DeVellis, 1991).

### 3.2 Inclusive education teachers’ attitude profiles

Table 2 shows the fit indices for the selection of the models estimated in this study using one to five latent profile solutions for teachers’ ATIE. The AIC, BIC, and ABIC values for the five profile solutions continued to decrease. The *p*-value of LMR for the fifth profile solution was not significant, which indicated that it was not significantly distinguishable from the fourth profile solution. The entropy value of the fourth profile solution was >0.80, which suggested that it had the highest classification accuracy. Overall, the fourth profile solution was superior to other solutions in this study.

**Table 2:** Comparisons of model fit indices for latent profiles of attitudes toward inclusive education.

No. of profiles	No. of free parameters	AIC	BIC	ABIC	*p*LMR	*p*BLRT	Entropy
1	6	8,770.66	8,799.94	8,780.88	-	-	-
2	10	8,304.06	8,352.86	8,321.10	0.000	0.000	0.66
3	14	8,133.84	8,202.15	8,157.68	0.001	0.000	0.75
4	18	8,030.58	8,118.40	8,061.24	0.002	0.000	0.82
5	22	7,952.27	8,059.62	7,989.75	0.388	0.000	0.77

**Figure 1:**
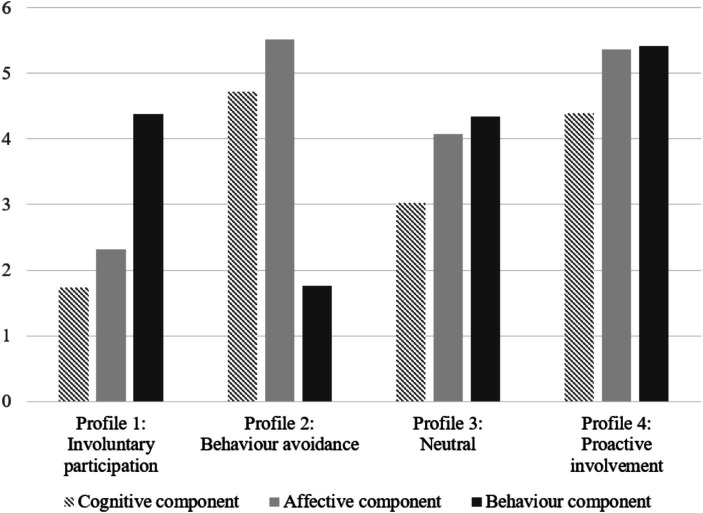
The four-profile solution of attitudes toward inclusive education.

The latent means and standard errors for the three ATIE components in the four-profile solution are presented in Table 3 and illustrated in Figure 1. Profile 1 (*n* = 76) was labeled *involuntary participation* because teachers belonging to this profile showed the lowest scores for the cognitive and affective components, suggesting that these teachers unwillingly participated in work related to inclusive education. Profile 2 (*n* = 11) was labeled *behavior avoidance* because teachers belonging to this profile scored highest for the cognitive and affective components but scored lowest on the behavioral component, indicating that although these teachers were in favor of inclusive education, they did not take action. Profile 3 (*n* = 497) was labeled *neutral* because it was characterized by moderate levels for all three attitude components. Profile 4 (*n* = 388) was labeled *proactive involvement* because it was characterized by high levels for all three attitude components.

**Table 3:** Means and standard errors for the four profiles of attitudes toward inclusive education.

	Profile 1: Involuntary participation	Profile 2: Behavior avoidance	Profile 3: Neutral	Profile 4: Proactive involvement
	*M* (*SE*)	*M* (*SE*)	*M* (*SE*)	*M* (*SE*)
Cognitive component	1.74 (0.18)	4.72 (0.35)	3.02 (0.07)	4.39 (0.11)
Affective component	2.32 (0.23)	5.52 (0.15)	4.08 (0.11)	5.37 (0.04)
Behavior component	4.38 (0.21)	1.76 (0.20)	4.34 (0.06)	5.41 (0.05)

### 3.3 Predictors of inclusive education teachers’ attitude profiles

Table 4 presents the multinomial logistic regression results concerning the influence of demographic factors (i.e., gender, teaching experience, subject taught, and in-service training) on the likelihood of attitude profile membership. Results showed significant differences in terms of in-service training (*χ*^2^ = 40.35, *p* < 0.001) and teaching experience (*χ*^2^ = 10.20, *p* < 0.05). Specifically, teachers who had participated in in-service training about inclusive education were approximately twice (OR = 1.75) more likely to belong to the *neutral* profile (profile 3) relative to the *involuntary participation* profile (profile 1) than those who had not participated in such training. Trained teachers were approximately four times (OR = 3.72) more likely to belong to the *proactive involvement* profile (profile 4) relative to the *involuntary participation* profile (profile 1) than untrained teachers. Trained teachers were simultaneously twice (OR = 2.13) as likely to belong to the *proactive involvement* profile (profile 4) relative to the *neutral* profile (profile 3) than untrained teachers. In addition, teachers with 1–15 years of teaching experience were about 1.5 times (OR = 1.46) more likely to belong to the *proactive involvement* profile (profile 4) relative to the *neutral* profile (profile 3) than those with >16 years of teaching experience. There were no significant gender or subject taught differences among the four profiles.

**Table 4:** Multinomial logistic regression results of the influence of demographic factors on profiles of attitudes toward inclusive education.

	Profile 2 vs. Profile 1			Profile 3 vs. Profile 1			Profile 4 vs. Profile 1		
	Coeff.	*SE*	OR	Coeff.	*SE*	OR	Coeff.	*SE*	OR
Gender	−0.22	0.79	0.80	−0.49	0.33	0.61	−0.42	0.34	0.65
Teaching	−1.13	0.72	0.32	−0.27	0.25	0.77	0.11	0.26	1.12
Subject	−1.14	0.72	0.32	−0.18	0.27	0.83	−0.28	0.28	0.75
Training	1.29	0.72	3.65	0.56*	0.25	1.75	1.31***	0.26	3.72

	Profile 3 vs. Profile 2			Profile 4 vs. Profile 2			Profile 4 vs. Profile 3		
	Coeff.	*SE*	OR	Coeff.	*SE*	OR	Coeff.	*SE*	OR
Gender	−0.27	0.74	0.76	−0.20	0.74	0.82	0.07	0.20	1.07
Teaching	0.86	0.69	2.36	1.24	0.69	3.44	0.38**	0.14	1.46
Subject	0.96	0.68	2.61	0.86	0.68	2.36	−0.10	0.15	0.90
Training	−0.73	0.69	0.48	0.02	0.69	1.02	0.75***	0.15	2.13

Profile 1 = involuntary participation; Profile 2 = behavior avoidance; Profile 3 = neutral; Profile 4 = proactive involvement; Coeff., coefficient; SE, standard error of the coefficient; OR, odds ratio; Teaching, teaching experience; Subject, subject taught; Training, in-service training related to inclusive education; *p < 0.05, **p < 0.01, ***p < 0.001.

### 3.4 Inclusive education teachers’ attitude profiles and organizational commitment

The ANOVA results indicated significant differences in all five organizational commitment components among the four ATIE profiles (Table 5). The *post hoc* analysis showed that teachers belonging to the *involuntary participation* profile scored significantly higher on maladaptive commitments (i.e., choice and economic commitments) than those belonging to the other three profiles. Teachers belonging to the *proactive involvement* and *behavior avoidance* profiles showed higher levels of adaptive commitments (i.e., affective, normative, and ideal commitments) than those belonging to the *involuntary participation* and *neutral* profiles.

**Table 5:** Differences in organizational commitment across the four profiles of attitudes toward inclusive education.

	Affective commitment	Normative commitment	Ideal commitment	Choice commitment	Economic commitment
	*M* (*SD*)	*M* (*SD*)	*M* (*SD*)	*M* (*SD*)	*M* (*SD*)
Profile 1	4.04 (0.09)	4.04 (0.09)	3.88 (0.10)	2.74 (0.13)	3.44 (0.13)
Profile 2	4.64 (0.23)	4.46 (0.24)	4.60 (0.25)	2.49 (0.34)	2.79 (0.34)
Profile 3	3.94 (0.04)	3.86 (0.04)	3.73 (0.04)	2.22 (0.05)	2.94 (0.05)
Profile 4	4.50 (0.04)	4.39 (0.04)	4.32 (0.04)	1.77 (0.06)	2.83 (0.06)
*F*	40.17***	34.61***	38.10***	20.75***	6.50***
*η*^2^	0.11	0.10	0.11	0.06	0.02
*Post hoc*	4 > 1; 4 > 3; 2 > 3	4 > 1; 4 > 3	4 > 1; 4 > 3; 2 > 3	1 > 3 > 4;	1 > 3; 1 > 4

Profile 1 = involuntary participation, Profile 2 = behavior avoidance, Profile 3 = neutral, Profile 4 = proactive involvement; ***p < 0.001.

## 4 Discussion

The four objectives of this study were achieved. First, in terms of the psychometric properties of the Revised-MATIES, both the exploratory factor analysis and confirmatory factor analysis results supported the three-component ATIE model (Mahat, 2008). Moreover, the reliability of the Revised-MATIES was higher than in studies that adopted the MATIES (Mahat, 2008; Štemberger and Kiswarday, 2018; Butakor et al., 2020). Therefore, the Revised-MATIES is a validated, reliable instrument that can be used to measure teachers’ ATIE in future studies.

Second, based on the three components (i.e., cognitive, affective, and behavioral) of the Revised-MATIES, four teachers’ ATIE profiles were identified via LPA: *involuntary participation*, *behavior avoidance*, *neutral*, and *proactive involvement*. According to Tein et al. (2013), Howard et al. (2016), and Ferguson et al. (2020), the lower values of AIC, BIC, and ABIC, the significant *p* values of LMR and BLRT, and the entropy values greater than 0.80 indicated adequate model fit indices for the best profile solution. In the current study, the four-profile solution met the above statistical criteria, suggesting that the identified four profiles were appropriate.

Among the four profiles, the *neutral* and *proactive involvement* profiles are similar to the two ATIE types explored in studies adopting variable-centered approaches: that is, neutral (Galović et al., 2014) and positive (Štemberger and Kiswarday, 2018), respectively. Unlike the results of variable-centered approaches, both the other two profiles (i.e., *involuntary participation* and *behavior avoidance*) showed opposite scores for the three ATIE components. Teachers belonging to the *involuntary participation* profile scored lower on the cognitive and affective components but scored higher on the behavioral component. This result indicated that although the teachers took action in implementing inclusive education, they did not believe that students with SEN should receive education in regular education schools and did not truly accept these students in their classrooms. Similar results were obtained in qualitative studies (Tiwari et al., 2015). One possible reason for this finding could be that teachers have no choice over the assignment of students with SEN to their classrooms because schools should not reject these students according to the zero rejection principle (Turnbull, 2005). Therefore, teachers must undertake the relevant tasks even though they are not prepared cognitively or affectively. Another possible reason that has been widely documented in the literature is that despite a great deal of time invested, students with SEN still cannot make desirable academic progress or reduce their challenging behaviors. Therefore, their teachers feel frustrated and doubt the effectiveness of inclusive education (Yeo et al., 2014; Tiwari et al., 2015).

In contrast to the *involuntary participation* profile, teachers belonging to the *behavior avoidance* profile scored higher on the cognitive and affective components but scored lower on the behavioral component. This result echoes the previous findings that teachers are in favor of inclusive education but have many concerns about its implementation (Yada and Savolainen, 2017). One of the major concerns is teachers’ lack of competence in teaching students with SEN (Forlin et al., 2008). For example, some Chinese teachers really want to teach students with SEN but are afraid of acting ‘with good intentions but doing something wrong’ (Jia and Santi, 2020) because they have no prior experience and relevant skills in teaching these students. Examination-oriented culture is another major concern. That is, teachers have no extra time and energy to consider the needs of students with SEN because they have to focus on their more able students to compete against other schools for better performance in exams (Jia and Santi, 2020). Therefore, teachers’ insufficient confidence and unfavorable school culture may lead to their avoiding taking action to implement inclusive education.

Another point concerning the characteristics of the ATIE profiles deserving special attention is that the scores of the cognitive component were lower than those of the affective component across all four profiles. This could potentially be attributed to the cultural context of Chinese society. China has actively advocated inclusive education in policy and practice since 2010. However, the teacher education system has not kept pace with the development of inclusive education. Previous research has pointed out that inclusive education curricula have not been widely integrated into training programs for pre-service and in-service teachers (Xu and Malinen, 2015; Xie et al., 2022). This resulted in current regular school teachers generally lacking scientific and systematic cognition in inclusive education and students with SEN. Nevertheless, deeply influenced by Confucian ideology, Chinese society generally displays inclusive and accepting attitudes toward disability (Deng and Su, 2012). This is because benevolence, a key concept of Confucian culture, encourages society to care for and assist people with disabilities (Deng and Su, 2012). Therefore, it is likely that although teachers may have inadequate knowledge and skills to educate students with SEN, they still emotionally support these students.

Third, in-service training about inclusive education and teachers’ teaching experience were the two major factors that statistically predicted teachers’ profile membership. With respect to in-service training, teachers with training experience were more likely to belong to the *involuntary participation*, *neutral*, and *proactive involvement* profiles, with the possibility of membership in each profile increasing in order. The differences among the three profiles are mainly reflected in the cognitive and affective components (see Figure 1); therefore, the above findings imply that in-service training could enhance teachers’ understanding of inclusive education and acceptance of students with SEN. This would allow teachers to belong to more desirable ATIE profiles (e.g., *proactive involvement*). However, the *behavior avoidance* profile was not predicted by in-service training when it was compared with the other profiles. One possible reason for this finding is that the in-service training received did not effectively improve teachers’ skills in implementing tasks related to inclusive education because the key difference between the *behavior avoidance* profile and other profiles was the teachers’ scores on the behavioral component of ATIE (see Figure 1). This result is in line with previous findings that many mainland Chinese in-service training programs focus on teaching inclusive education principles and theories as well as describing the characteristics of students with SEN rather than teaching operational strategies for inclusive education (Xu and Malinen, 2015).

With respect to teaching experience, the result showed that teachers with 1–15 years of teaching experience were more likely to belong to the *proactive involvement* profile relative to the *neutral* profile than those with >16 years of teaching experience. This result is consistent with the finding in variable-centered studies that teachers with less teaching experience tend to held more positive ATIE (Butakor et al., 2020). One of the probable explanations may be found in Hargreaves’ study, which explored how teachers at different career stages responded to educational change (Hargreaves, 2005). Hargreaves revealed that compared with teachers in a later career stage (> 20 years of experience), teachers in their early and mid-careers were more enthusiastic, more willing to accept new challenges, and showed more positive attitudes toward educational change (Hargreaves, 2005). Therefore, teachers with less teaching experience tend to accept students with SEN and be willing to explore ways to implement inclusive education.

Finally, teachers belonging to the four ATIE profiles showed significant differences in their commitment to inclusive education schools. Specifically, teachers belonging to the *involuntary participation* profile showed the highest levels of maladaptive organizational commitments (i.e., economic and choice commitments). If teachers are forced to undertake work related to inclusive education, they tend to continue working for their school because of economic rewards and limited job choices. In terms of adaptive organizational commitments (i.e., affective, normative, and ideal commitments), teachers belonging to the *proactive involvement* and *behavior avoidance* profiles showed higher levels than teachers belonging to the other two profiles. The similarities of the *proactive involvement* and *behavior avoidance* profiles are that teachers belonging to these two profiles scored higher on the cognitive and affective components of ATIE. This result implies that recognizing the meaning of inclusive education and accepting students with SEN could help teachers establish their conducive organizational commitment to schools. This finding could be interpreted by the person-organization value congruence theory (Amos and Weathington, 2008), which argues that higher levels of congruence between individuals and their organization are an important precondition for positive psychosomatic outcomes (e.g., organizational commitment). Therefore, if teachers’ educational ideals are in line with their schools’ mission to develop inclusive education, these teachers would be more likely to perceive their obligation to devote themselves to implementing inclusive education (i.e., normative commitment), pursue their occupational goals concerning inclusive education (i.e., ideal commitment), and establish an emotional attachment to their schools (i.e., affective commitment).

Somewhat unexpectedly, teachers belonging to the *neutral* profile (not those in the *involuntary participation* or *behavior avoidance* profiles) showed the lowest levels of adaptive commitments. One of the possible explanations is that these teachers are bystanders in their schools’ inclusive education reform. Regardless of their cognitive, affective, or behavioral aspects, teachers do not truly engage in inclusive education. To some extent, they may think that their school’s mission to develop inclusive education has nothing to do with them. Therefore, it is hard for them to establish adaptive commitments to inclusive education schools. Among the 972 participants in this study, 497 teachers were categorized in the *neutral* profile, suggesting that a possible solution for improving the effectiveness of inclusive education could be to change teachers’ neutral ATIE.

## 5 Conclusions, contributions, implications, and limitations

### 5.1 Conclusion

This study identified four profiles for teachers’ ATIE: *involuntary participation*, *behavior avoidance*, *neutral*, and *proactive involvement*. In-service training and teaching experience play key roles in determining teachers’ profile membership. Teachers in the *behavior avoidance* and *proactive involvement* profiles tend to show adaptive organizational commitment to inclusive education schools compared with teachers belonging to the other profiles.

### 5.2 Theoretical contributions

This study has three major theoretical contributions. First, this study modified and verified the MATIES in the Chinese inclusive education context. The Revised-MATIES is a validated, reliable instrument to measure teachers’ ATIE; therefore, it can be used in future studies. Second, to the best of the authors’ knowledge, it is the first time that teachers’ ATIE has been examined through a person-centered approach (e.g., LPA), which extends the understanding of the complex ATIE held by different teachers. Third, this study enriches the ATIE and organizational commitment literature.

### 5.3 Practical implications

In addition to the above theoretical contributions, this study provides practical implications for in-service teacher training programs, inclusive education school principals, and teachers of students with SEN. Teachers with in-service training experiences tend to belong to desirable ATIE profiles; therefore, in-service teacher training programs related to inclusive education should be organized more frequently. The training programs could contain three modules based on the three ATIE components: enhancing teachers’ understanding of inclusive education, expanding teachers’ knowledge about students with SEN, and improving teachers’ skills (e.g., behavior management and collaboration) related to implementing inclusive education.

Given that teachers with less teaching experience are more likely to belong to the *proactive involvement* profile, inclusive education school principals could assign important roles (e.g., activity organizers) to these teachers. Moreover, as teachers belonging to the *involuntary participation* and *neutral* profiles tend to show maladaptive commitment to their schools, school principals could strengthen their advocacy of inclusive education to help these teachers understand the school’s mission to develop inclusive education.

Considering teachers of students with SEN, those who belong to the *involuntary participation* and *neutral* profiles could improve their understanding of inclusive education (e.g., through in-service training or self-learning) to prepare both cognitively and affectively to teach students with SEN. Teachers belonging to the *behavior avoidance* profile could develop relevant skills through, for example, attending relevant in-service training programs and collaborating with special educators.

### 5.4 Limitations and future research directions

Four major limitations of this study must be addressed in future research. First, the present participants were in-service teachers working in inclusive education schools in Beijing, China; therefore, the findings may not be generalizable to teachers in other cultures. Future research could replicate this study to explore teachers’ ATIE profiles in other parts of China and worldwide.

Second, the four profiles were identified in this study using a quantitative method. However, the explanations for why some teachers belong to a certain profile (e.g., *involuntary participation*) were not based on their voice as participants. Future studies could adopt a mixed-methods design where a quantitative analysis (e.g., LPA) is used to identify the teachers’ ATIE profiles and a qualitative analysis (e.g., semi-structured interview) is used to explore the reasons why some teachers belong to a certain profile.

Third, when examining the probable determinants of teachers’ attitude profile membership, this study found that teaching experience and in-service training were important. However, the differentiation of the *behavior avoidance* profile from the remaining three profiles was not predicted by any factor selected in this study. Future studies could use broader factors (e.g., students’ type of disability) to explore other determinants of teachers’ attitude profile membership.

Fourth, the methodological limitation of this study may cause potential bias in the results. On the one hand, all instruments employed in this study were self-report inventories, which may result in social desirability bias. Future studies could adopt other-report inventories to measure teachers’ ATIE and organizational commitment. On the other hand, this study adopted a cross-sectional research design, so that statistical causality cannot be established. Future research could use a longitudinal design to examine more robust causal relationships among teachers’ demographic factors, ATIE profiles, and organizational commitment.

## Data availability statement

The raw data supporting the conclusions of this article will be made available by the authors, without undue reservation.

## Ethics statement

The studies involving humans were approved by the Human Research Ethics Committee (HREC) of the University of Hong Kong. The studies were conducted in accordance with the local legislation and institutional requirements. The participants provided their written informed consent to participate in this study.

## Author contributions

YD: Conceptualization, Investigation, Methodology, Writing – original draft. WY: Formal analysis, Validation, Writing – review & editing. L-fZ: Conceptualization, Supervision, Writing – review & editing. ZX: Conceptualization, Formal analysis, Investigation, Writing – original draft, Writing – review & editing.

## Footnotes

^1^ https://www.wjx.cn/
